# Effect of nurses’ perception to workplace civility climate on nurse–physician collaboration

**DOI:** 10.1002/nop2.666

**Published:** 2020-11-04

**Authors:** Eman Kamel Hossny, Hanaa Esmail Sabra

**Affiliations:** ^1^ Nursing Administration Department Faculty of Nursing Assiut University Assiut Egypt; ^2^ Nursing Administration Department Faculty of Nursing South Valley University Quena Egypt

**Keywords:** civility, climate, collaboration, nurses, physicians

## Abstract

**Aims:**

To assess the effect of nurses’ perception to workplace civility climate on nurse–physician collaboration and determine whether the largest contributor to changing the climate of the workplace are the employees or top management.

**Design:**

This study used non‐experimental descriptive cross‐sectional study design, which using purposive sample (139 nurses) working at Health Insurance Hospital.

**Method:**

The current study data were collected by using self‐administered questionnaires including three tools: (a) a structured interview questionnaire includes personal data; (b) perceived workplace civility climate scale (15 items); and (c) Nurse Physician Collaboration Scale (27 items).

**Results:**

Main result of this study reveals that there was a positive statistically significant correlation between workplace civility climate and collaboration (0.208^*^). Studied nurses were perceived top management with the high score (87.5) than employees (65.2).

**Conclusion:**

Workplace civility climate was demanded for well collaboration between nurses and physicians to provide high‐quality services; when nurses and physicians have increase chance to work in civility climate, combining with response to incivility and low intolerance for it, they will be more collaborated. Recommendation: Policies and procedures are very important in healthcare settings to address uncivil actions and establish a civilized climate.

## 1 INTRODUCTION

It is expected that the nurse will demonstrate professional behaviours when communicating with the patient, colleague, doctor or manager. Civility represents societal standards and guidelines that must be followed for effective communication with others and a good work environment improves nurses for providing patient‐focused care (Al‐Shehry et al., 2019). The civility and respect with which hospital units’ deal create an atmosphere of high mood and job satisfaction. In addition, patients are satisfied with the care provided when there is collaboration between nurses and doctors (Al‐Hussami et al., 2017). The climate of civility in the workplace has an impact on collaboration between healthcare professionals especially nurses and doctors, and few studies have examined this issue in this regard. Accordingly, nurses and physicians, collaborative relationships are the cornerstone in patient therapy (Aghamohammadi et al., 2019).

The collaboration and relationship between nursing staff and physicians have fluctuated until now. Several studies indicate a low level of cooperation between nursing staff and physicians (Elham & El‐Hanafy, 2018). Others discovered that the level of cooperation on the part of working nurses was high compared with that of physicians (Melkamu et al., 2020). Elsous et al., (2017) defined collaboration between nurses and physicians as “nurse and physician are performing in cooperation, their responsibilities are shared on resolving difficulties and decisions which are taken to frame and perform plan for patient care.”

Managers and nurses are responsible in the clinical setting for maintaining civility in the workplace. It is a legal, ethical and moral responsibility for managers to create safe work environments that lead to the health and well‐being of nurses and other healthcare team members, patients and patient families (ANA, 2015).

### 1.1 Background

Historically, according to Patterson, (2016) the concept of “civility” comes from the ideas of citizenship, civilization and the city. Civilized people are those who fit to live in cities, and the idea of civility is necessary because it helps people to live in close relationships with one another and gives a basic basis for cooperation, collective and society. Civility also governs the expression of our attitudes. The attitudes here include a series of emotions such as disgust, disdain, love, anger and fear. It also extends to include judgments, symptoms, hopes and desires.

Accordingly, civility in the workplace stems from this broad historical concept of civility, as civility is the behaviour that helps maintain standards of mutual respect at work, so workplace civility climate is a direct expansion of a safety climate with less damage. The civility climate in the workplace was defined as the employees' perception of how to manage practices, procedures, policies and rules to keep the workplace civil. Management can take actions to stimulate a harmless work setting by establishing policy and procedure that direct worker behaviours associated with safety. Supervisors, as liaison personnel between management and practical nurses, are central to distribute civil behaviours from top to bottom (Cortina et al., 2017).

Therefore, together with the employees, management plays an important role in cultivating a climate of civility in the workplace. In addition to this, nurses' self‐awareness of policies within the framework is required to deal with indifferent behaviours (ANA, 2015). The climate of constructive civility in the workplace adds to good cooperation, communication, patient safety and quality of care. Based on it, collaboration between doctors and nurses must be integrated into the healthcare system to support the quality of correct healthcare (Hossny & Ahmed, 2019). In addition to improving patient outcomes and satisfaction of nurses in different healthcare settings (Elsous et al., 2017).

Civility in the workplace needs training, which is the tool to help reduce the incidence of abuse and improve personal relationships (Walsh & Magley, 2018). Organizational support in the units for initiatives based on correcting the culture of incivility will further define goals which can have a positive impact on employee engagement, team member cooperation, relationships and patient care (Jun, 2017). Workplace incivility can be disruptive to the overall function of clinical units; however, instituting a zero‐tolerance policy for incivility will begin a positive change and lead to better outcomes for our staff and our patients (Green, 2019).

Nurses' perception of civility or lack of civility in the workplace differs according to some social and demographic factors through the nursing profession such as gender and experience in practicing nursing, age, marital status and level of education. The nurse's job may be associated with increased physical and psychological workload and ageing, and therefore, the longer work period has been associated with higher values of requests and desired changes, lower levels of support, luxury and control (Kowalczuk & Krajewska‐Kułak, 2015). Therefore, the civilized working relationship between the doctor and the nurse increases the nurses association with her work and loyalty and vice versa, as studies have shown that the unsatisfactory professional relationship between the nurse and the doctor partly contributed to the nurses’ shortage and leaving their work (Elsous et al., 2017).

### 1.2 Significance of the study

Healthcare services have become complicated, and cooperation between nurses and doctors is a global problem facing healthcare providers. Previous studies have recommended the need for more studies to examine various ways to improve cooperation between nurses and doctors and increase incentives to create a civil work climate. Based on that and after reviewing the national and international literature on cooperation between nurses and doctors and the climate of civility in the workplace, the researcher will study the effect of nurses' perception to workplace civility climate on collaboration between nurses and physicians. In addition to determining whether the largest contributor to changing the civility climate in the workplace are the employees or top management.

### 1.3 Study hypothesis

H1 The nurses who will perceive workplace civility climate have a positive effect on nurse–physician collaboration.

H2 The nurses who will perceive top management are the largest contributor to changing the civility climate in the workplace.

### 1.4 Aims of the study

To assess the effect of nurses’ perception to workplace civility climate on nurse–physician collaboration and to determine whether the largest contributor to changing the civility climate in the workplace is the employees or top management.

## 2 METHODS

### 2.1 Design

A non‐experimental descriptive cross‐sectional study design was used.

### 2.2 Study setting

The study was conducted on all units (35) in the Health Insurance Hospital (AlMabra Hospital) with a capacity of 397 beds, and this hospital belongs to the Ministry of Health. The hospital consists of two separate buildings: one for education and the other to provide healthcare services.

### 2.3 Study subject

A sample of qualified nurses (*N* = 139) who are on duty above‐mentioned study settings at the time of the study who are voluntary agreed to participate in this study after clarification the main purpose to them and who have job experience more than two years. A purposive sample was calculated based on this equation: *n* = *N* ÷ 1 + *N* (*e*) ^2^. Here, “*n*” = sample size, “*N*” = the total number of nurses and “*e*” = coefficient factor (0.005) (Yamane, 1967).

### 2.4 Tools of the study

The current study data were collected by using self‐administered questionnaires including three tools; first one is a structured interview questionnaire. This questionnaire was developed by the researcher after reviewing of the related literature and includes personal data related to age, gender, educational qualification, marital status, department and years of experience. Second one is perceived workplace civility climate scale (PWCCS). It is developed by Ottinot (2008) and used to measure nurses perception of workplace civility climate. It includes 15 items in a three dimensions: intolerance for incivility (six items), response (four items) and policies and procedures (five items). Previous internal reliability for the policies and procedures dimension (*α* = 0.78), the response dimension (*α* = 0.66) and the intolerance for incivility dimension (*α* = 0.76) was all adequate. The current internal reliability for the same categories the policies and procedures dimension (*α* = 0.78.4), the response dimension (*α* = 0.68) and the intolerance for incivility dimension (*α* = 0.79) was all adequate. The researcher show to put an open‐ended question based on recommendation from experts to ask nurses determine which level (management or employees) is more contributed for change civility climate at workplace from their perception. Scoring system for this scale as following; Participants rated items using a five‐point Likert scale from 1 = strongly disagree to 5 = strongly agree. Scale scores were calculated for each dimension, with higher scores on the response and policies/procedures dimensions indicating higher levels of perceived workplace civility climate (PWCC) along each dimension. Intolerance for incivility items was reverse‐scored, with higher scores indicating employees perceived the organization as having greater intolerance for incivility.

Third one is Nurse–Physician Collaboration Scale (NPCS) . It was developed in Japan by (Ushiro, 2009). It measures the actual collaborative actions between nurse and physician. NPCS includes 27 items is divided into three categories: Sharing of the clinical patient information; Decision‐making process; and Nurse physician cooperation, and the questionnaire was translated using the back translation method to ensure an accurate reportage of the original meaning. The researcher translates the questionnaire into Arabic, using the same method (back translation) to ensure accurate meaning, and then, juries was done with three experts from professors in faculty of nursing, after that internal consistency was done to ensure interrelation between items. Its scoring system as following: items of this scale are rated on a 5‐point Likert scale ranging from strongly agree = 1—strongly disagree = 5 (from 5–1) with the lowest score meaning the greatest degree of collaboration.

### 2.5 Procedures

The procedures of this study included three stages: preparatory, pilot study and field work. Preparatory phase started at the beginning of January 2019 to the end of February 2019. It includes reviewing related national and international literature in scientific journals, textbooks and scientific websites on the Internet. Tools were translated into Arabic and retranslated into English for accuracy.

#### 2.5.1 Validity of the study

The content validity and psychometric analyses of the questionnaires were assessed by five professors in the field of education in administration department and community department at Assiut University (face validity through a jury).

#### 2.5.2 Pilot study

A pilot study was carried out in February 2019, and it was done on 10% of study subject that included (14 nurse) before starting actual data collection for the purpose of ascertain the clarity, applicability of study tools and time estimation for filling it which ranged between about 30–40 min. Studied nurses participated in the pilot study not excluded from the study because of no modification was done in the study tools. Internal consistency was measured using Cronbach's alpha coefficients (0.82–0.90), thus indicating a high degree of reliability.

#### 2.5.3 Fieldwork

After ensuring the clarity and understandability of the study tools, the actual data collection took about two months started in March 2019 and ended in April 2019. The researchers met with all participated nurses and explained to them the purpose of the study, and they were asked for oral consent to participate in this study; then, the researcher distributed the questionnaire form to them individually, and in groups, the researcher stays with participants for any clarification. Data were collected in different shifts according to work schedule of each unit.

#### 2.5.4 Ethical considerations

Research proposal was approved from Ethical Committee at the Faculty of Nursing, Assiut University. There is no risk for study participants during application of the research. The study was following common ethical principles in clinical research. Oral agreement was taken from the participants in this study. Study participants have the right to refuse or to participate and/or withdraw from the study without any rational at any time. Confidentiality and anonymity were assured.

### 2.6 Analysis

All analysis was performed with the IBM SPSS 20.0 software. The data tested for normality using the Anderson–Darling test and for homogeneity variances prior to further statistical analysis. Categorical variables were described by number and per cent, while continuous variables were described by mean and standard deviation. A two‐tailed *p* < .05 was considered statistically significant.

## 3 RESULTS

**Figure 1:**
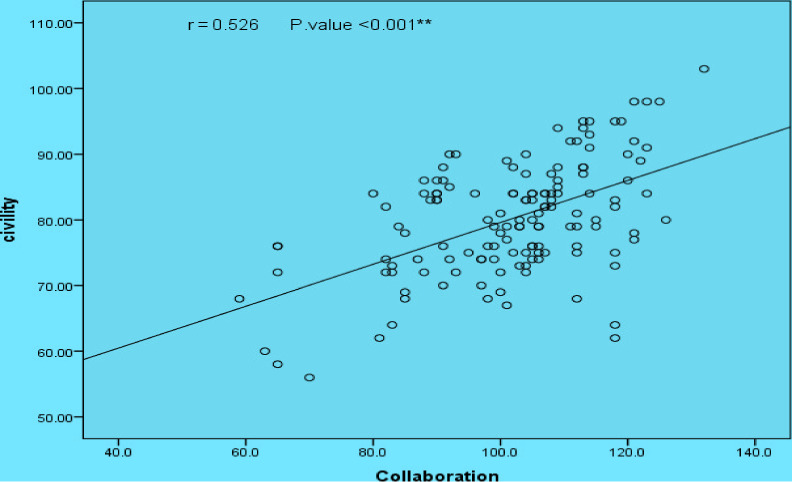
Linier regression between perceived workplace civility climate (PWCC) and Nurse–Physician Collaboration Scale (NPCS) as perceived by studied nurses. Statistically Significant Correlation at *p*. value  .01

Figure 1 Linier regression between PWCC and Nurse–Physician Collaboration Scale (NPCS) as perceived by studied nurses, as shown in this figure a highly statistically significant relation between civility and collaboration.

**Figure 2:**
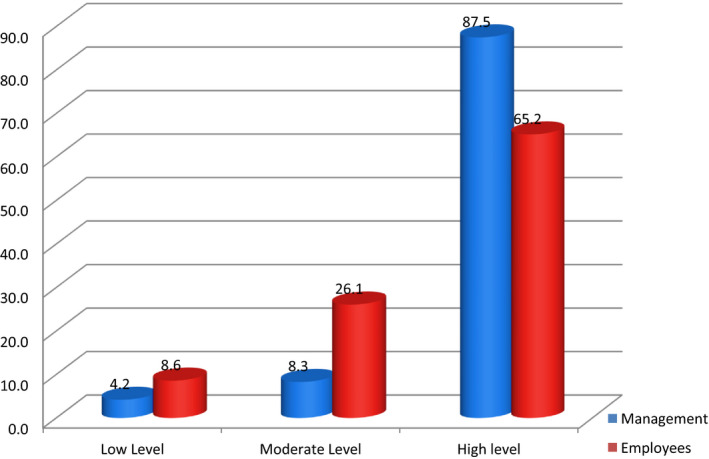
Management and employees levels regarding to contribution for changing organizational climate as perceived by studied nurses

Figure 2 demonstrated studied nurses perceived management with the highest score (87.5) of contribution for changing civility climate than line of employees. Also, it is important to note that both (management and employees) in the high level.

Table 1 reveals the distribution of personal data of the studied nurses, more than half (56.8%) of studied nurses have more than 30 years old, and more than two‐third of them (64.8%) have experience less than 10 years. Most (84.2%) are female, distributed in medical, orthopaedic, surgical, paediatric and intensive care units (ICUs) (30.9%, 15.8%, 14.4%, 12.9% and 12.9%), respectively, and more than half (53.2%) of them graduated have diploma qualification, and least qualification (7.9%) have bachelor of nursing, lastly most of them are married (82.7%).

**Table 1:** Distribution of personal data of the studied nurses (*N* = 139))

Personal data	No	%
Age		
Less than 30 year	60	43.2
More than 30 years	79	56.8
Gender		
Male	22	15.8
Female	117	84.2
Education level		
Bachelor of Nursing	11	7.9
Nursing Diploma	74	53.2
Nursing technical institutes	54	38.8
Marital status		
Single	24	17.3
Married	115	82.7
Years of experience		
Less than 10 years	89	64.8
More than 10 years	50	35.2
Departments		
Surgical	20	14.4
Chest	7	5.2
Paediatric	18	12.9
Medical	43	30.9
Maternity	11	7.9
ICU	18	12.9
Orthopaedic	22	15.8

Table 2 Max score, range, mean and standard deviation of main items of PWCCS. The highest mean score of workplace civility climate scale related to intolerance for incivility (II) (20.37 *SD* 3.51) followed by response (R) (18.68 *SD* 2.73) and the lowest mean score related to policies and procedures (PP) (17.83 *SD* 2.85).

**Table 2:** Max score, range, mean and standard deviation of main items of perceived workplace civility climate scale (PWCCS)

PWCC	Max score	Range	Mean ± *SD*
II	30	10–30	20.37 ± 3.51
R	25	13–25	18.68 ± 2.73
PP	25	10–24	17.83 ± 2.85
TC	80	47–68	56.88 ± 4.92

Abbreviations: II, Intolerance for incivility; PP, Policies and procedure; R, Response; TC, Total civility.

Table 3 Max score, range, mean and standard deviation (*SD*) of main items of Nurse–Physician Collaboration Scale (NPCS) . Sharing of clinical patient information (SCPI) has the high max score, range, mean and *SD* (50, 21–50, 37.71 *SD* 5.83), respectively, followed by nurse physician cooperation (NPC) (45, 18–45, 33.43 *SD* 5.46) and the lowest one score related to decision‐making process (DMP) (40, 12–40, 30.73 *SD* 5.11).

**Table 3:** Max score, range, mean and standard deviation (*SD*) of main items of Nurse–Physician Collaboration Scale (NPCS)

NPCS	Max Score	Range	Mean ± *SD*
SCPI	50	21–50	37.71 ± 5.83
DMP	40	12–40	30.73 ± 5.11
NPC	45	18–45	33.43 ± 5.46
TC	135	59–132	101.86 ± 14.26

Abbreviations: DMP, decision‐making process; NPC, nurse physician cooperation; SCPI, sharing of clinical patient information; TCL, total collaboration.

Table 4 Correlation between items of PWCC scale and items of NPCS. The data show that there is a significant positive correlation (0.208^*^) between (PWCC) scale and (NPCS), in general. Regarding to correlation between the main items of both scales, the data reveal that there are highly statistical significant positive correlation between response (R) and all items of (NPCS) (0.383^**^, 0.270^**^, 0.291^**^), respectively. The same condition present between the item of policies and procedures (PP) and all items of (NPCS) (0.388^**^, 0.318^**^, 0.380^**^), respectively. On contrary, there are statistical significant negative correlation between intolerance for incivility (II) and all items of (NPCS) (−0.365‐^**^, −0.199‐^*^, −0.287‐^**^), respectively.

**Table 4:** Correlation between items of perceived workplace civility climate (PWCC) scale and items of Nurse–Physician Collaboration Scale (NPCS)

(NPCS) Items	(PWCC) Items			
	II	R	PP	TCI
SCPI	−0.365**	0.383**	0.388**	0.176*
DMP	−0.199*	0.270**	0.318**	0.191*
NPC	−0.287**	0.291**	0.380**	0.177*
TCO	−0.330**	0.365**	0.418**	0.208*

* Correlation is significant at the 0.05 level (two‐tailed).

** Correlation is significant at the 0.01 level (two‐tailed).

Table 5 Correlation coefficient between socio‐demographic data PWCC scale and NPCS. Data in this table reveal positive significant correlation between age and PWCC (*r* = 0.268^**^). Regarding to Nurse–Physician Collaboration Scale (NPCS) , there is no statistically significant correlation between it and all items of socio‐demographic data.

**Table 5:** Correlation coefficient between socio‐demographic data, perceived workplace civility climate (PWCC) scale and Nurse–Physician Collaboration Scale (NPCS)

Correlation coefficient		Age	Gender	Education level	Years of experience	Departments	M. status
PWCC	R	0.268**	−0.044	−0.105	−0.130	−0.142	0.135
	P	0.001	0.611	0.217	0.117	0.096	0.113
NPC	r	0.081	0.043	0.026	0.070	−0.074	−0.028
	P	0.345	0.613	0.760	0.418	0.386	0.744

* Correlation is significant at the 0.05 level (two‐ailed).

** Correlation is significant at the 0.01 level (two‐tailed).

## 4 DISCUSSION

In this study, the researchers will discuss two important points: one relates to assessing the effect of nurses’ perception of civility in the workplace on the collaboration between the nurse and the physician, and the second relates to determining whether the largest contributors to changing the workplace climate are the employees or the management, both of them represent the aims of this study. According to data in our study as revealed in Figure 2, the nurses realize that management and employees were at the same high level, but top management has the highest score (87.5) in changing the workplace civility climate. From the researcher's point of view, these data are valuable and draw the attention of healthcare managers who are the head of the hospital/organization as it has the greatest responsibility towards developing the climate of the civil workplace through policies, procedures, rules and regulations. It sets to create a framework for the levels of interaction between healthcare personnel. Especially among nurses and physicians, hypothesis 2 in this regard was confirmed.

According to Ottinot (2010) organizations can establish a climate of workplace civility in several ways. First, the organization should adopt official policies and procedures for addressing workplace incivility and verbal abuse. Second, management, though line supervisors, can state and emphasize to employees how coworkers are to be treated.

Nurses and physicians in the organization represent the largest number of its employees, and cultivating the climate of civility in the workplace is inevitable for healthy collaborative relationship. According to results in this study, as showed in Figure 1, there was a high statistical significant relation between PWCC and NPC. Also, the correlation between PWCC and NPC in Table 4 revealed that, there was a positive statistically significant correlation (0.208^*^) between them in general. These results accordance with Laschinger et al. (2012) that nurses who work in the workplace with character of civility climate enable them to practice nursing well and have the character of a cooperative and civil relationship among their employees.

In addition to that, in Table 4, it was observed that there was a positive correlation of high statistical significance between response to incivility (R) and all elements of the (NPC) especially with exchange of patient clinical information and physician–nurse cooperation (383** and 291**), respectively. This means that nurses who work in the workplace respond to uncivil behaviours, and they are unlikely to be exposed to life pressures at work (Laschinger et al., 2012); accordingly, cooperation and sharing information are better. These findings were confirmed by the results of the current study that the highest mean score is related to clinical patient information sharing (SCPI) and then physician–nurse cooperation in Table 3. According to Mathur (2011) the high agreement related to collaboration between nurses and physicians appears in different ways like exchange clinical information about patient, nature of cooperation between them and decision‐making.

Moreover, there was a strong significant positive correlation present between policies and procedures (pp) and all elements of the (NPC). Policies and procedures (PP) are inevitable for establishing the climate of civility which the Joint Commission since (2008) urged all organizations to adopt a zero‐tolerance policy towards uncivil behaviours in the workplace and encouraged the development of policies and codes of conduct. These results confirmed hypothesis 1 that the nurses who will perceive workplace civility climate have a positive effect on nurse‐physician collaboration.

On other hand, there is statistically significant negative correlation between intolerance for incivility (II) and all items of the NPCS. This means that collaboration between nurses and physicians has been affected negatively when organization intolerance for incivility increase. According to results revealed in this study in Table 2, it was noted that intolerance for incivility (II) has the highest mean scores and this result was in line with the result of the study that was conducted at Mansoura University, Egypt, by Sleem and Seada (2017) that found that a high mean score is associated with intolerance for incivility. Based on this result, the administrators should provide a space for healthy collaboration than pressure results from increased intolerance for incivility.

The data in this study in Table 1 showed that more than half of the nurses were over 30 years old and graduated from nursing diploma (56.8% and 53.2%), respectively. And more than two‐thirds of them (64.8%) had less than 10 years of experience. The correlation between personal data and both of NPC and PWPCC in Table 5 revealed that, there was no statistical significant relation appears between them, and the only strong statistically significant relation was between WPCC and age (268**). This means that with increase nurses' age, their perception to workplace civility climate will increase, which more than half of them in this study have more than 30 years old (56.8%). Also, according to study results conducted by Sleem and Seada (2017) reveals that no statistical relation between workplace civility climate and personal data except only with gender in their study.

### 4.1 Limitation of the study

The researchers have some limitations, such as this study conducted only on nurses; however, physicians should be involved in the future studies. Also, this study conducted in one ministry of health hospital, for generalization of data so in the future studies many hospitals in Assiut city can be involved.

## 5 CONCLUSION

The current study is one of the unique to connect collaboration between nurses and physicians with workplace civility climate in this essential part of the healthcare employees. Civility climate is very important for healthy collaborative relationship between nurses and physicians; when nurses and physicians have increased chance to work in civility climate, combined with response to incivility and low intolerance for it, they will be more collaborated. Civility climate was not created alone, and it needs top management to cultivate it through establishing policies and procedures and the need for line employees. Personal data did not have significant effect on both workplace civility climate and collaboration between nurses and physicians except age with civility.

## 6 RECOMMENDATIONS

### 6.1 At management level

Hospital management and their employees are look like a human body, working in a harmonizing manner; management established assertive policies and procedures for control over healthcare professionals’ behaviours in the frame of establishing workplace civility climate, set a role model of pleasant and civil behaviours using the supervisors and head nurses as a management agents. In the same line, hospital employees are the hands of the management, and they follow the managerial instructions. Also, it is important of preparing seminars for physician and nurse about meaning of collaboration between them for the purpose of fit and civilian work climate, plus positive reflection on patient care.

### 6.2 At educational level

It is necessary that nursing education curricula included topics related to civility climate in their curricula. It is also useful to have undergraduates' role play different situations. It has been observed that new graduates are able to switch uncivil behaviours into a new suitable way through using role play.

## CONFLICT OF INTEREST

No conflict of interest present.

## Data Availability

No data available online.

## References

[nop2666-bib-0001] Aghamohammadi, D. , Dadkhah, B. , & Aghamohammadi, M. (2019). Nurse‐physician collaboration and the professional autonomy of intensive care units staff nurses. Indian Journal of Critical Care Medicine, 23(4), 178–181. 10.5005/jp-journals-10071-23149 31130789 PMC6521826

[nop2666-bib-0002] Al‐Hussami, M. , Al‐Momani, M. , Hammad, S. , Maharmeh, M. , & Darawad, M. (2017). Patients' perception of the quality of nursing care and related hospital services. Health and Primary Care, 1(2), 1–6. 10.15761/HPC.1000110

[nop2666-bib-0003] Alshehry, A. S. , Alquwez, N. , Almazan, J. , Namis, I. M. , Moreno‐Lacalle, R. C. , & Cruz, J. P. (2019). Workplace incivility and its influence on professional quality of life among nurses from multicultural background: A cross‐sectional study. Journal of Clinical Nursing, 28(13–14), 2553–2564. 30811737 10.1111/jocn.14840

[nop2666-bib-0004] American Nurses Association . (2015). Incivility, bullying and workplace violence. Retrieved from www.nursingworld.org/practicepolicy/nursing‐excellence/officialposition‐statements/id/incivilitybullying‐and‐workplace‐violence

[nop2666-bib-0005] Cortina, L. , Kabat‐Farr, D. , Magley, V. J. , & Nelson, K. (2017). Researching rudeness: The past, present and future of the science of incivility. Journal of Occupational Health Psychology, 22, 299–313. 10.1037/ocp0000089

[nop2666-bib-0007] Elham, Y. , & El‐Hanafy, Y. (2018). Nurse physician work‐related relationship as perceived by both of them. Egyptian Nursing Journal, 15, 188–195.

[nop2666-bib-0008] Elsous, A. , Radwan, M. , & Mohsen, S. (2017). Nurses and physicians attitudes toward nurse‐physician collaboration: A survey from Gaza Strip, Palestin. Nursing Research and Practice, 2017, Article ID 7406278, 7 pp. 10.1155/2017/7406278 PMC5343283 28326194

[nop2666-bib-0010] Green, C. (2019). Workplace incivility: Nurse leaders as change agents PERFORMANCE POTENTIAL. Nursing Management, 50(1), 51–53. 10.1097/01.NUMA.0000550455.99449.6b 30601385

[nop2666-bib-0011] Hossny, E. , & Ahmad, E. (2019). Effect of negotiation on collaboration between nurses and physicians at South valley University Hospital, Egypt. International Journal of Novel Research in Healthcare and Nursing, 6(2), 1499–1508.

[nop2666-bib-0012] Joint Commission . (2008). Behaviors that undermine a culture of safety. Sentinel event alert. Retrieved from www.jointcommission.org/assets/1/18/SEA_40.PDFGoogleScholar 18686330

[nop2666-bib-0013] Jun, S. (2017). Mediating effect of social capital between transformational leadership behavior and organizational citizenship behavior in hospital nurses. Journal of Korean Academy of Nursing Administration, 23(5), 558–566.

[nop2666-bib-0014] Kowalczuk, K. , & Krajewska‐Kułak, E. (2015). Influence of selected socio demographic factors on psychosocial workload of nurses and association of this burden with absenteeism at work. Medycyna Pracy, 66(5), 615–624. 10.13075/mp.5893.00076 26647980

[nop2666-bib-0015] Mathur, P. (2011). Conflict management proficiency and stress of managers in instrumentation industry. Elixir Management Arts, 41, 6055–6056.

[nop2666-bib-0016] Melkamu, E. , Woldemariam, S. , & Haftu, A. (2020). Inter‐professional collaboration of nurses and midwives with physicians and associated factors in JIMMA University specialized teaching hospital, Jimma, south West Ethiopia, cross sectional study. BMC Nursing, 19:33, 2–9. 32351325 10.1186/s12912-020-00426-w PMC7183687

[nop2666-bib-0017] Ottinot, R. C. (2008). The development and validation of the perceived workplace civility climate scale. *A published thesis submitted in partial fulfillment of the requirements for the degree of Master of Arts Department of Psychology College of Arts and Sciences University of South Florida*.

[nop2666-bib-0018] Ottinot, R. C. (2010). A multi‐level study investigating the impact of workplace civility climate on incivility and employee well‐being. University of South Florida, pp (1:97). Corpus ID: 114070403.

[nop2666-bib-0019] Patterson, A. M. (2016). Construct Analysis of Civility in the Workplace. *Published Doctor Thesis of Philosophy in Psychology*. University of Guelph.

[nop2666-bib-0020] Sleem, W. , & Seada, A. (2017). Role of workplace civility climate and workgroup norms on incidence of incivility behavior among staff nurses. International Journal of Nursing Didactics, 7(6), 34–43. 10.15520/ijnd.2017.vol7.iss6.230.34-43

[nop2666-bib-0021] Spence Laschinger, H. K. , Leiter, M. P. , Day, A. , Gilin‐Oore, D. , & Mackinnon, S. P. (2012). Building empowering work environments that foster civility and organizational trust testing an intervention. Nursing Research, 61(5), 316–325. 10.1097/NNR.0b013e318265a58d 22935768

[nop2666-bib-0022] Ushiro, R. (2009). Nurse‐physician collaboration scale: Development and psychometric testing. Journal of Advanced Nursing, 65, 1497–1508. 19635097 10.1111/j.1365-2648.2009.05011.x PMC2738564

[nop2666-bib-0023] Walsh, B. , & Magley, V. (2018). Workplace civility training: Understanding drivers of motivation to learn. The International Journal of Human Resource Management, 10.1080/09585192.2018.1441164

[nop2666-bib-0024] Yamane, T. (1967). Statistics: An introductory analysis (2nd ed., pp. 180–181). Harper & Row.

